# The effects of intensive home treatment on self-efficacy in patients recovering from a psychiatric crisis

**DOI:** 10.1186/s13033-020-00426-y

**Published:** 2021-01-06

**Authors:** Ansam Barakat, Matthijs Blankers, Jurgen E Cornelis, Nick M Lommerse, Aartjan T F Beekman, Jack J M Dekker

**Affiliations:** 1 Department of Research, Arkin Mental Health Care, Klaprozenweg 111, 1033 NN Amsterdam, The Netherlands; 2 Department of Psychiatry Amsterdam UMC/VUmc, Amsterdam Public Health research institute Amsterdam UMC, Amsterdam, The Netherlands; 3 Trimbos-Institute, Netherlands Institute of Mental Health and Addiction, Utrecht, The Netherlands; 4 Department of Psychiatry Amsterdam UMC/AMC, Amsterdam Public Health research institute Amsterdam UMC, Amsterdam, The Netherlands; 5 Department of Emergency Psychiatry, Arkin Mental Health Care, Amsterdam, The Netherlands; 6 Department of Research and Innovation, GGZ InGeest Specialized Mental Health Care, Amsterdam, The Netherlands; 7 Department of Clinical Psychology, Vrije Universiteit Amsterdam, Amsterdam Public Health research institute Amsterdam UMC, Amsterdam, The Netherlands

**Keywords:** Intensive home treatment, Self-efficacy, Emergency psychiatry, Randomised controlled trial

## Abstract

**Background:**

This study evaluated whether providing intensive home treatment (IHT) to patients experiencing a psychiatric crisis has more effect on self-efficacy when compared to care as usual (CAU). Self-efficacy is a psychological concept closely related to one of the aims of IHT. Additionally, differential effects on self-efficacy among patients with different mental disorders and associations between self-efficacy and symptomatic recovery or quality of life were examined.

**Methods:**

Data stem from a Zelen double consent randomised controlled trial (RCT), which assesses the effects of IHT compared to CAU on patients who experienced a psychiatric crisis. Data were collected at baseline, 6 and 26 weeks follow-up. Self-efficacy was measured using the Mental Health Confidence Scale. The 5-dimensional EuroQol instrument and the Brief Psychiatric Rating Scale (BPRS) were used to measure quality of life and symptomatic recovery, respectively. We used linear mixed modelling to estimate the associations with self-efficacy.

**Results:**

Data of 142 participants were used. Overall, no difference between IHT and CAU was found with respect to self-efficacy (B = − 0.08, SE = 0.15, *p* = 0.57), and self-efficacy did not change over the period of 26 weeks (B = − 0.01, SE = 0.12, t (103.95) = − 0.06, *p* = 0.95). However, differential effects on self-efficacy over time were found for patients with different mental disorders (F(8, 219.33) = 3.75, *p* < 0.001). Additionally, self-efficacy was strongly associated with symptomatic recovery (total BPRS B = − 0.10, SE = 0.02, *p* < 0.00) and quality of life (B = 0.14, SE = 0.01, *p* < 0.001).

**Conclusions:**

Although self-efficacy was associated with symptomatic recovery and quality of life, IHT does not have a supplementary effect on self-efficacy when compared to CAU. This result raises the question whether, and how, crisis care could be adapted to enhance self-efficacy, keeping in mind the development of self-efficacy in depressive, bipolar, personality, and schizophrenia spectrum and other psychotic disorders. The findings should be considered with some caution. This study lacked sufficient power to test small changes in self-efficacy and some mental disorders had a small sample size.

*Trial registration* This trial is registered at Trialregister.nl, number NL6020.

## Background

For many years, hospitalisation has been the standard care modality for patients experiencing a severe psychiatric crisis. More recently, Intensive Home Treatment (IHT), sometimes called Crisis Resolution Home Treatment [1], has become available as an outpatient alternative.

IHT is a brief 6 week intervention, and is offered to patients with a wide-ranged variety of psychiatric disorders [1, 2]. IHT is provided by a multidisciplinary team, which aims to resolve a psychiatric crisis by providing intensive care in the patient’s home setting. A crisis can be defined as “a perception or experience of an event or situation as an intolerable difficulty that exceeds the person’s current resources and coping mechanisms” [3]. IHT teams offer psychiatric treatment, emotional and practical support, and psycho-education for the patient and their relatives. Moreover, they focus on improving problem-solving and everyday skills [4, 5].

Among other things, IHT aims to empower patients in crisis and improve the patients’ confidence in their ability to take control of their functioning and social environment, which is strongly related to the concept of self-efficacy [4–6]. According to Bandura, self-efficacy is an individual's belief in their capacity to execute behaviours necessary to produce specific performance attainments [7–9].

In chronic conditions, such as most psychiatric disorders, self-efficacy plays a considerable role in recovery and is also associated with quality of life [10–13]. It is crucial to empower patients during a crisis with the belief in their ability to cope with their symptoms, as the crisis unfolds [14, 15]. Furthermore, in patients experiencing stressful life events [14] decreased self-efficacy is shown to be related to suicidality [16], relapse and recurrence of depression [17], social anxiety disorder [18] and psychosis [19]. Self-efficacy has also been found to be directly associated with treatment outcomes, or treatment outcomes were mediated by self-efficacy [20, 21].

To our best knowledge, only a few studies on IHT mention the promotion of self-efficacy as a factor of empowerment. Qualitative studies by Moran et al. [22] and Winness et al. [23] explored the views of mental health professionals, managers and patients on the use, principles and implementation of IHT. Both studies showed that patients and professionals advocated improving self-efficacy by IHT. There is a need for quantitative examination of the effect of IHT on self-efficacy and the subsequent impact of self-efficacy on clinical outcomes, such as quality of life (QoL) and symptomatic recovery. Managing a crisis at home provides opportunities to enhance a person’s self-efficacy through the acquisition of new skills and insights that subsequentially remain valuable in future crises [24, 25]. Therefore, it is essential to understand and expand our knowledge on the impact of self-efficacy and how it relates to patients’ well-being and symptomatic recovery in the case of IHT.

Using the data from a randomised controlled trial (RCT), the present study aims to test the hypothesis that receiving IHT results in a stronger increase of self-efficacy compared to care as usual (CAU) during a 26 weeks follow-up period. Moreover, we were interested whether patients with different psychiatric disorders showed a similar development in self-efficacy across the above-mentioned treatment groups. Additionally, we examined the associations between self-efficacy and both symptomatic recovery and QoL, as we hypothesised an association between self-efficacy and these outcome measures.

## Methods

The study was nested in a large Zelen design RCT assessing the effects of IHT compared to CAU. The Medical Ethics Committee of VU University Amsterdam (METc VUmc) approved the study (#NL55432.029.16). This study has been performed in accordance with the ethical standards laid down in the 1964 Declaration of Helsinki and its later amendments. The protocol of the RCT has been published previously [26]. In brief; patients were recruited by IHT teams and from psychiatric wards between November 2016 and October 2018. Patients included were 18 to 65 years of age, experiencing an acute psychiatric crisis for which hospitalisation was deemed necessary by a psychiatrist, classified according to the DSM-IV-TR or DSM-V with at least one axis I or II disorder, and residents of Amsterdam, The Netherlands. Patients were excluded if they were homeless, were primary classified with a substance use disorder for which detoxification was needed, had intellectual disabilities, lacked basic understanding of the Dutch language, received (Flexible) Assertive Community Treatment care, or had previously received IHT. Patients who met the study criteria were pre-randomised to IHT or CAU using the Zelen double consent open-label design [27]. The applied allocation ratio was 2:1 for reasons of staff and facility capacity. Before participation, assessment of the patient’s capability to consent for research took place; patients deemed incapable to consent were not included. Participants were interviewed by trained researchers as soon as informed consent was given and had follow-up interviews at 6, 26 and 52 weeks. For this study, the baseline, 6 and 26 weeks follow-up measurements were used in the analyses.

### Treatment groups

The IHT group of this study comprises all patients who were randomised to IHT and who received this treatment immediately after allocation or, when hospitalised, as soon as discharge was considered. IHT teams provide intensive care at least twice a week and continue until the crisis is resolved for an average duration of 6 weeks.

The CAU group comprises patients who were allocated to specialised mental health care hospital or other less intensive outpatient care (i.e. two times a week or less). The actual treatment received depended on the severity of the symptoms, presence of danger, housing and availability of support system.

### Measurements

Self-efficacy was measured using the Mental Health Confidence Scale (MHCS) at baseline, 6, 26 and 52 weeks. This questionnaire is recommended for mental health care patients due to its good psychometric qualities and compactly formulated items referring to specific daily situations [28]. The MHCS is a self-report questionnaire consisting of 16 statements that assess self-efficacy beliefs across three dimensions [29]. According to a study by Castelein et al.[28], the psychometric internal consistency of the total score of all subscales had a Cronbach’s α of 0.94, the subscales optimism (six items), coping (seven items) and advocacy (three items) had a Cronbach’s α of 0.88, 0.87 and 0.76 respectively. Participants were requested to rate the given statements on a six point scale ranging from 1, ‘very unconfident’ to 6, ‘very confident’. Higher scores on the MHCS indicate higher self-efficacy.

At baseline, several socio-demographic and background variables were collected; such as age, gender, country of birth, education level and employment status. Psychiatrists classified psychiatric disorders by using the Diagnostic and Statistical Manual of Mental Disorders (DSM-IV-TR or DSM-V depending on the year of inclusion). QoL was assessed using the 5-dimensional EuroQol instrument (EQ-5D-5L). For the EQ-5D-5L composite scores were calculated using the Dutch tariff [30]. The EQ-5D has a Cronbach’ s α value of 0.63 in patients with schizophrenia in an acute psychiatric setting [31]. Higher composite scores on the EQ-5D-5L indicate better QoL. Recovery defined as a symptomatic outcome was measured using the Brief Psychiatric Rating Scale (BPRS). This instrument has an internal consistency ranged from 0.69 to 0.74. The 24-item BPRS has symptoms grouped into subscales: Positive symptoms, Negative symptoms, Depression and Anxiety, Disorganisation, and Total BPRS [32]. Higher scores on the BPRS are indicative of poorer mental health and thus lower symptomatic recovery.

### Data analysis

For all analyses, linear mixed modelling with a two‐level structure (repeated measures, within patients) was used. All statistical analyses were performed using IBM SPSS Statistics software version 26.0 for Windows and all two-sided statistical test were performed with a significance level of α = 0.05. As we were interested in the effect of IHT and CAU on self-efficacy in patients who received this treatment, we only analysed the data of patients who actually received IHT or CAU in this study (per-protocol analysis). A sensitivity analysis was performed in which the data from all included patients were analysed (intention-to-treat analysis). For the secondary analysis of this study we calculated that at least 106 patients were needed to detect a self-efficacy difference of 0.25 standard deviations (Cohen’s d = 0.25) with a power of 0.80 and a conventional two-sided alpha level of 0.05.

To assess the difference between IHT and CAU with respect to all self-efficacy dimensions over time, the basic model comprised: Treatment, a Time variable as indicator for the longitudinal development, and a two-way interaction term (Time*Treatment). The treatment groups were represented by a dichotomous variable: CAU (0) versus IHT (1), time was added as dichotomous variable representing: 6 weeks (0) and 26 weeks (1). A random intercept was included in the model. We also accounted for the self-efficacy baseline measurement by adding the baseline measure as a covariate in each model.

Next, the development of self-efficacy in different psychiatric disorders across treatment groups was explored. The model comprised: Treatment, psychiatric disorders, Time and a two-way interaction term (Diagnosis*Treatment) testing if participants in the treatment groups with different psychiatric disorders had different self-efficacy scores. Subsequently, in the second model a two-way interaction term tested whether self-efficacy developed differently over time for participants with different psychiatric disorders (Diagnosis*Time). Psychiatric disorders were limited to the four most frequently diagnosed disorders of the participants, i.e. depressive, bipolar, personality and schizophrenia spectrum and other psychotic disorders. All other disorders were combined into one category “other”. Time was added as a numeric variable representing: baseline (0), 6 weeks (1) and 26 weeks (2). Post hoc linear mixed modelling analysis was conducted if self-efficacy developed differently over time for participants with different psychiatric disorders. To account for multiple comparisons the Bonferroni correction was used.

Lastly, the association between self-efficacy and both symptomatic recovery and QoL was tested. The basic model comprised Positive symptoms, Negative symptoms, Depression and Anxiety, Disorganisation, Total BPRS or EQ-5D-5L scores, time variable, and a two-way interaction term (Time*Self-efficacy). This two-way interaction term tested if change in self-efficacy over time was associated with change in symptomatic recovery or QoL. BPRS and EQ-5D-5L scores were represented as a continuous variables. Time was added as a numeric variable: baseline (0), 6 weeks (1) and 26 weeks (2). A random intercept was added to account for different baseline response values.

## Results

### Participants

Of the 246 patients, 48 patients gave permission to use their medical records but did not complete the self-report instruments (Additional file 1: Figure S1). Hence, no self-efficacy scores were available for these participants. Of the remaining 198 participants, 56 (28%) participants did not receive IHT or CAU as randomised and were therefore excluded from the per-protocol analyses, while they were included in the intention-to-treat sensitivity analyses. The per-protocol analyses were based on the data of 142 participants of whom 49 (34.5%) and 93 (65.5%) were randomised to the CAU and IHT condition, respectively.

### Socio-demographics

As shown in Table 1, the mean age of the participants was 41.51 (SD = 12.00), 81 (57.0%) were female and 78 (55.3%) were in paid employment. Of the participants, 58 (41.1%) were highly educated, 116 (81.7%) were born in the Netherlands and 78 (55.3%) were living with others at baseline. Participants had various primary diagnoses: 35 participants were diagnosed with depressive disorders (25%), 35 with bipolar disorders (25%), 38 with schizophrenia spectrum and other psychotic disorders (27.1%), 10 with personality disorders (7.1%), 4 with substance abuse (2.9%), 14 participants had other disorders (10.0%), and 4 (2.9%) were undiagnosed at the time of inclusion. Quality of life as measured using the EQ-5D-5L was similar for IHT (mean = 0.76, SD = 0.25) and CAU (mean = 0.78, SD = 0.26) (t (128) = 0.37, *p* = 0.71). The total BPRS scores of participants who received IHT and CAU varied somewhat (IHT: mean = 1.81, SD = 0.39; CAU: mean = 1.69, SD = 0.36), but not significantly (t (128) = − 1.69 *p* = 0.09). We found that neither the self-efficacy score (*p* = 0.63) nor the self-efficacy subscales differed significantly between the IHT and CAU condition at baseline (Table 2).

**Table 1:** Per-protocol participants’ characteristics across the treatment groups at baseline

Characteristics	IHT		CAU		Total	
	N	%	N	%	N	%
Gender, female	59	63.4	22	44.9	81	57.0
Employed, yes	56	60.9	22	44.9	78	55.3
Country of birth, the Netherlands	73	78.5	43	87.8	116	81.7
Education						
Low	9	9.8	5	10.2	14	9.9
Middle	43	46.7	26	53.1	69	48.9
High	40	43.5	18	36.7	58	41.1
Living with others, yes	56	60.9	22	44.9	78	55.3
Most common disorders						
Depressive	25	27.2	10	20.8	35	25.0
Bipolar	23	25.0	12	25.0	35	25.0
SSOPD	24	26.1	14	29.2	38	27.1
Personality	6	6.5	4	8.3	10	7.1
Substance abuse	2	2.2	2	4.2	4	2.9
Other	9	9.8	5	10.4	14	10.0
Undiagnosed^†^	3	3.3	1	2.1	4	2.9

	Mean	SD	Mean	SD	Mean	SD
Age	40.72	12.09	43.02	11.82	41.51	12.00
Total BPRS	1.81	0.39	1.69	0.36	1.77	0.38
EQ-5D-5L	0.77	0.25	0.79	0.26	0.78	0.26

*IHT* Intensive Home Treatment, *CAU* Care as Usual, *SD* standard deviation, *SSOPD* Schizophrenia Spectrum and Other Psychotic Disorders, *BPRS* the Brief Psychiatric Rating Scale, *EQ-5D-5L* 5-dimensional EuroQol instrument

^†^Participants were undiagnosed at the time of the inclusion

**Table 2:** Self-efficacy scores of intensive home treatment and care as usual

Self-efficacy dimensions		N	Time	IHT			CAU			t	DF	*p*
				Mean	95% CI		Mean	95% CI				
					Lower	Upper		Lower	Upper			
Optimism		126	Baseline	4.03	3.73	4.32	4.30	3.91	4.68	1.10	124	0.27
		125	6 weeks	4.23	3.98	4.48	4.25	3.93	4.56	0.07	123	0.94
		112	26 weeks	4.12	3.83	4.38	4.23	3.84	4.62	0.50	110	0.62
Advocacy		125	Baseline	4.61	4.35	4.87	4.43	4.07	4.78	−0.86	123	0.39
		125	6 weeks	4.65	4.43	4.87	4.65	4.40	4.91	0.01	123	0.99
		112	26 weeks	4.69	4.50	4.88	4.71	4.38	5.03	0.70	110	0.94
Coping		124	Baseline	3.67	3.39	3.95	3.78	3.36	4.20	0.44	122	0.66
		125	6 weeks	4.04	3.80	4.28	3.88	3.53	4.23	-0.74	123	0.46
		111	26 weeks	3.86	3.62	4.11	4.01	3.66	4.37	0.72	109	0.47
Self-efficacy		124	Baseline	3.99	3.74	4.24	4.09	3.73	4.46	0.48	122	0.63
		125	6 weeks	4.23	4.01	4.44	4.17	3.89	4.44	-0.34	123	0.74
		112	26 weeks	4.11	3.90	4.33	4.20	3.85	4.54	0.44	110	0.66

The outcome based on a per-protocol analysis

*Self-efficacy* the total of all self-efficacy dimensions, *CAU* Care as usual, *IHT* intensive home treatment, *N* number of participants included in the analysis, *CI* confidence interval, *t* T-test value, *DF* degrees of freedom

### Intensive home treatment and self-efficacy

Table 3 shows the results of the linear mixed modelling analyses of the associations of time and condition on self-efficacy. We found that the main effect of Time on self-efficacy was not significant (B = − 0.01, SE = 0.12, t (103.95) = − 0.06, *p* = 0.95), indicating that overall, self-efficacy did not change over the period of 26 weeks. We neither found a significant main effect of Treatment (B = − 0.08, SE = 0.14, t (172.85) = − 0.55, *p* = 0.59) nor a significant Time*Treatment interaction effect (B = − 0.08, SE = 0.15, t (104.66) = − 0.57, *p* = 0.57). These results indicate that participants receiving IHT and CAU did not differ significantly with regards to their self-efficacy scores during the 26 weeks follow-up period. All results for the self-efficacy subscales were similar to the results for the self-efficacy total score. The outcome of the sensitivity analysis did not differ from the per-protocol analysis (Additional file 2: Table S1).

**Table 3:** Effect of intensive home treatment and care as usual on self-efficacy dimensions during 26 weeks

Linear mixed modelling analyses									
Self-efficacy dimensions	N	Main effects	B	95% CI		SE	DF	t	*p*
				Lower	Upper				
Optimism	116	Intercept	2.09	1.58	2.61	0.26	127.12	8.02	< 0.001
		Baseline Optimism	0.54	0.44	0.65	0.05	112.82	10.08	< 0.001
		Treatment	− 0.08	− 0.41	0.25	0.17	178.88	− 0.50	0.62
		Time	− 0.05	− 0.33	0.24	0.14	106.09	− 0.32	0.75
		Time*Treatment	− 0.08	− 0.44	0.27	0.18	106.79	− 0.46	0.65
Advocacy	116	Intercept	3.05	2.53	3.57	0.26	124.13	11.57	< 0.001
		Baseline Advocacy	0.38	0.27	0.48	0.05	105.68	7.03	< 0.001
		Treatment	− 0.12	− 0.44	0.20	0.16	197.72	− 0.74	0.46
		Time	0.04	− 0.28	0.36	0.16	102.04	0.23	0.82
		Time*Treatment	− 0.01	− 0.41	0.39	0.20	103.00	− 0.07	0.95
Coping	115	Intercept	2.15	1.65	2.64	0.25	128.64	8.60	< 0.001
		Baseline Coping	0.50	0.39	0.61	0.06	115.19	8.94	< 0.001
		Treatment	− 0.02	− 0.35	0.32	0.17	170.10	− 0.09	0.93
		Time	0.05	− 0.23	0.33	0.14	104.35	0.33	0.74
		Time*Treatment	− 0.15	− 0.50	0.20	0.18	104.65	− 0.85	0.40
Self-efficacy	115	Intercept	2.15	1.68	2.61	0.23	123.54	9.15	< 0.001
		Baseline Self-efficacy	0.53	0.43	0.63	0.05	112.67	10.30	< 0.001
		Treatment	− 0.08	− 0.36	0.20	0.14	172.85	− 0.55	0.59
		Time	− 0.01	− 0.24	0.22	0.12	103.95	− 0.06	0.95
		Time*Treatment	− 0.08	− 0.37	0.21	0.15	104.66	− 0.57	0.57

The outcome based on a per-protocol analysis, based on estimated marginal means

*Self-efficacy* the total of all self-efficacy dimensions, *N* number of participants included in the analysis, *B* estimated regression coefficient, *CI* confidence interval, *SE* standardised error, *DF* degrees of freedom, *t* T-test value

**Table 4:** Differences between psychiatric disorders across three time points

Disorder	Mean	95% CI		SE	DF	Post hoc analysis (*p* < 0.05)
		Lower	Upper			
Baseline						
Depression	3.23	2.90	3.57	0.17	223.84	1 < 2;1 < 3
Bipolar	4.73	4.40	5.06	0.17	223.24	2 > 1; 2 > 4
SSOPD	4.43	4.12	4.75	0.16	221.72	3 > 1; 3 > 4
Personality	3.36	2.72	4.01	0.33	222.68	4 < 2; 4 < 3
6 weeks						
Depression	3.93	3.59	4.26	0.17	229.28	-
Bipolar	4.54	4.21	4.87	0.17	228.42	-
SSOPD	4.55	4.24	4.86	0.16	217.17	-
Personality	3.68	3.06	4.31	0.32	204.65	-
26 weeks						
Depression	3.88	3.54	4.21	0.17	234.74	-
Bipolar	4.44	4.10	4.77	0.17	233.86	-
SSOPD	4.37	3.04	4.71	0.17	258.44	-
Personality	4.08	3.44	4.73	0.33	222.68	-

Included data according per-protocol analyses, based on estimated marginal means. The shown *p*-value account for multiple comparisons using the Holm-Bonferroni corrections

*SE* standardised error, *DF* degrees of freedom, *CI* confidence interval, *SSOPD* Schizophrenia Spectrum and Other Psychotic Disorders

Groups in post hoc analysis noted as: 1 = Depressive disorders, 2 = Bipolar disorders, 3 = SSOPD, 4 = Personality disorders

### Psychiatric disorders and self-efficacy

The four most frequently diagnosed psychiatric disorders in our sample were depressive disorders (n = 35, 25%), bipolar disorders (n = 35, 25%), schizophrenia spectrum and other psychotic disorders (n = 38, 27.1%) and personality disorders (n = 10, 7.1%). Based on the information in the electronic medical record of the participants diagnosed with bipolar disorder, 76% (n = 25, missing = 10) were in the manic phase of bipolar disorder at baseline.

We found a main effect of Diagnosis on self-efficacy (F (4, 124.51) = 7.09, *p* < 0.001), which means that the self-efficacy scores varied across the different disorders. The effect of the interaction term Diagnosis*Treatment was not significant (F (4, 124.54) = 0.15, *p* = 0.96), indicating that there was no difference in self-efficacy scores between IHT and CAU across psychiatric disorders.

**Fig. 1:**
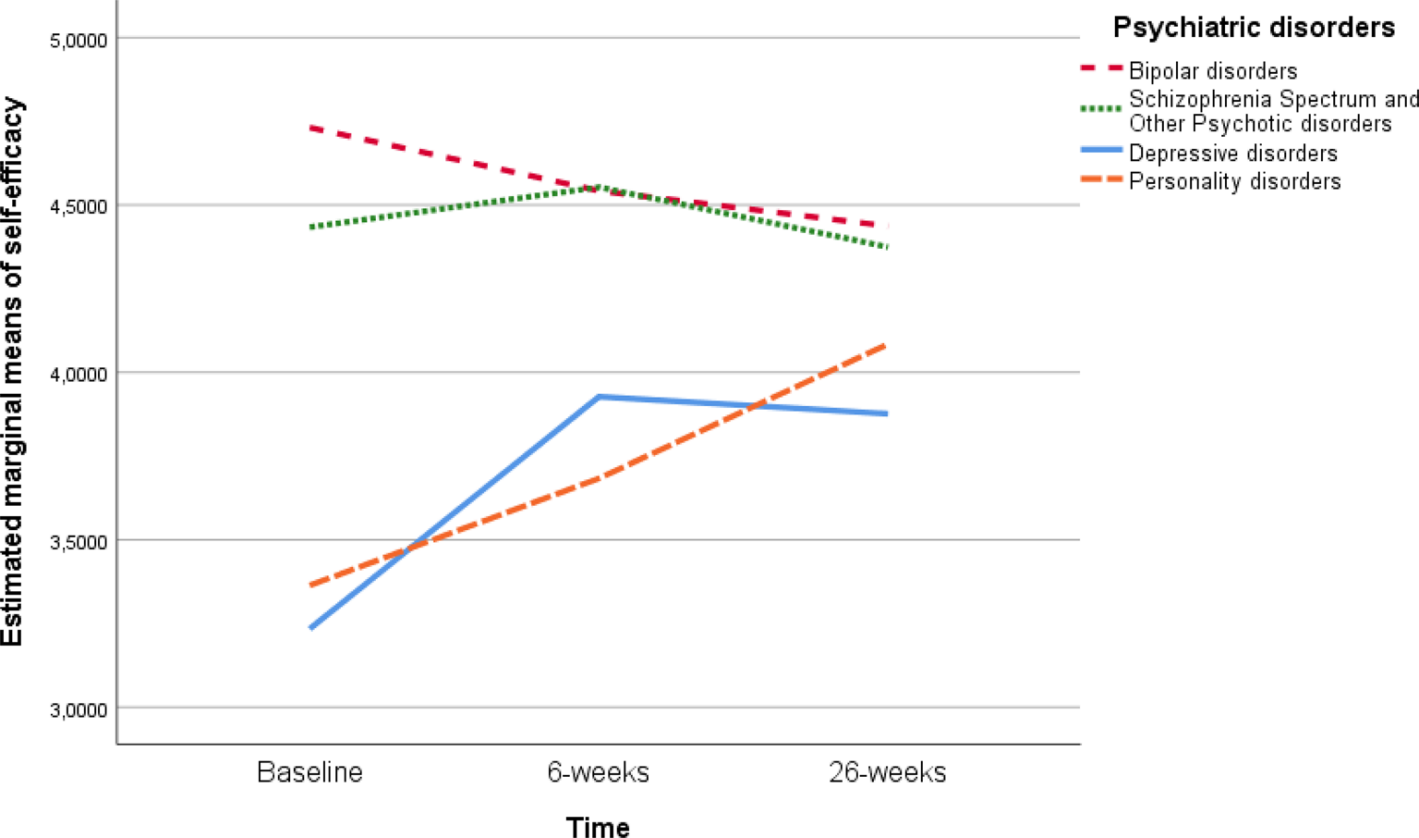
Longitudinal development in self-efficacy of diverse mental disorders

Next, we evaluated the development of self-efficacy over time for the different diagnostic groups. We found a main effect of Time on self-efficacy (F (2, 218.20) = 5.05, *p* = 0.007) and a significant Diagnosis*Time interaction (F (8, 219.33) = 3.75, *p* < 0.001). This indicates that the longitudinal trajectories of self-efficacy scores varied between the four psychiatric disorders during the 26 weeks follow-up period (Fig. 1).

In a post-hoc analysis, considering Bonferroni corrections, we examined the differences between the psychiatric disorders on self-efficacy scores across all three time points (Table 4). At baseline, self-efficacy scores of participants with bipolar or schizophrenia spectrum and other psychotic disorders were significantly higher compared to participants with depressive or personality disorders. At six and 26 weeks, no significant differences in self-efficacy were found between the psychiatric disorders.

We further examined the difference in self-efficacy scores for each of the psychiatric disorders across all three time points (Additional file 3: Table S2). For participants with a depressive disorder, self-efficacy scores at baseline were significantly lower compared to both six and 26 weeks (*p* < 0.001); for participants with a personality disorder, baseline scores were significantly lower compared to 26 weeks (*p* = 0.04).

### Symptomatic recovery and quality of life

Using mixed modelling, we examined the associations between self-efficacy and both symptomatic recovery and QoL. Linear mixed modelling analyses showed that Time was not significantly associated with total BPRS (B = 0.11, SE = 0.07 t (257.84) = 1.57, *p* = 0.12), which means that there was no unique time effect on psychiatric symptoms in this model. Self-efficacy (B = − 0.10, SE = 0.02, t (351.36) =− 4.66, *p* < 0.001) and the Time*Self-efficacy interaction term (B = − 0.06, SE = 0.02, t (257.49) = − 3.71, *p* < 0.001) were negatively associated with total BPRS score. This indicates that an increase in self-efficacy was associated with lower BPRS scores and thus with symptomatic recovery overall and during the follow-up period. The associations between self-efficacy and all BRPS subscales are shown in Additional file 4: Table S3.

Finally, we tested whether self-efficacy was associated with QoL. Self-efficacy was positively associated with EQ-5D-5L scores (B = 0.14, SE = 0.01, t (352.50) = 9.59, *p* < 0.001), indicating that higher self-efficacy was associated with higher QoL scores. We found no significant main effect of Time (B = 0.02, SE = 0.05, t (265.48) = 0.39, *p* = 0.69), nor a significant Time*Self-efficacy interaction effect (B < 0.01, SE = 0.01, t (264.27) = −0.34, *p* = 0.73).

## Discussion

The aim of this study was to test whether IHT results in a more substantial increase of self-efficacy compared to CAU among patients experiencing a severe psychiatric crisis. Based on the assumption that IHT contains elements related to the concept of self-efficacy namely to improve patient’s confidence in their ability to take control of their functioning and social environment, we hypothesised that receiving IHT would result in a stronger increase of self-efficacy compared to CAU. This study did not substantiate this hypothesis. In the present study, patients’ self-efficacy scores did not differ between the treatment groups IHT and CAU, and did not change over time. Next, we explored if self-efficacy varied for patients with different psychiatric disorders. We found that self-efficacy scores varied between depressive, bipolar, personality and schizophrenia spectrum and other psychotic disorders, overall and over time. Furthermore, we hypothesised an association between self-efficacy with symptomatic recovery or QoL. Our study partially confirmed this hypothesis; self-efficacy is associated with symptomatic recovery and with quality of life. In addition, changes in self-efficacy over time were associated with changes in symptomatic recovery, but not with changes in quality of life.

Improving self-efficacy is deemed important by patients and professionals and integral to the IHT model [22, 23]. This promotion of self-efficacy might be explained by the relationship between recovery and self-efficacy [15, 33], as the results of this study indicate. Nonetheless, it might not be reasonable or feasible to empower self-efficacy during an acute psychiatric crisis, since other problems may take priority at that moment of crisis. IHT is short-term, highly complex and contains a large number of varying components where the primary focus lies with, treatment of the crisis by prevention of danger, stabilisation, diagnosing the patient and subsequently starting a treatment. We may have to accept that targeting the improvement of self-efficacy will be addressed after the initial crisis and perhaps by follow-up treatment.

There might also be an intra- or interpersonal explanation as to why self-efficacy did not increase during our follow-up period. All patients were enrolled while they were experiencing a severe psychiatric crisis and thereafter were recovering from this crisis. Self-efficacy involves one’s perceived capabilities of successfully performing a specific behaviour to achieve a desired outcome, with the notice that the person must have the appropriate skills and stimulus for performance [9]. However, during a psychiatric crisis, appropriate skills like coping, advocacy and optimism are impaired [34]. Patients trying to recover from a severe event have doubt and fear of losing control whilst also dealing with stigma and doubt from their social system. All these effects from the crisis could undermine the recovery of self-efficacy. On the other hand, our results show that self-efficacy increases over time and is associated with symptomatic recovery. Symptomatic recovery might in turn lead to the recovery of quality of life [35, 36], therefore a longitudinal association between quality of life and self-efficacy might require a longer follow-up period. More research is needed to understand and unravel in which way psychiatric patients can empower self-efficacy after severe psychiatric crises.

An interesting finding in our study is the differential development of self-efficacy across different disorders. The results show that patients with depressive disorders increased their self-efficacy between baseline and both six and 26 weeks. Increase in self-efficacy has been found to be associated with less depressive symptoms and anxiety [37, 38], which was also established in our study. However, this increase in self-efficacy in patients with depressive disorders tended to decline slightly after six weeks. This could be an important finding as decreasing self-efficacy may be a risk factor of relapse [39, 40]. Notable to the above-mentioned findings, a bidirectional relation between psychopathology and self-efficacy is not excluded. Self-efficacy beliefs might be negatively affected by poor emotional well-being [8]. Patients with depressive and anxiety symptoms often show less effective emotion regulation, such as acceptance and problem solving suppression [41, 42], which in return increase poor emotional well-being [43] and thus might result in lower levels self-efficacy. Based on this and previous studies, the recommendation for mental health professionals is to give more attention to those specific patients to (possibly) prevent relapse.

In contrast to patients with depressive disorders, bipolar patients reported a decline in their self-efficacy over the 26 weeks follow-up period. At the baseline interview, which was conducted as soon as possible after the start of the initial crisis, patients reported high self-efficacy scores, possibly indicating a manic state of mind. Mania is suggested to be characterised by high levels of self-worth, self-confidence and personal value [44, 45] and a polar opposite of depression. As confirmed in our study during the crisis period. At follow-up, a decline in self-efficacy may indicate recovery from this manic episode. Lima and colleagues concluded in a recent review that patients with bipolar disorder show an increased negative and positive emotion reactivity after remission [45]. They also highlighted the variety of emotion regulation strategies that patients with bipolar disorders employ. However, little is known about self-efficacy levels during a relapse and remission period. Regarding the development of self-efficacy in patients diagnosed with schizophrenia spectrum and other psychotic disorders, we did not find a significant difference over time in this study, although visual inspection of the data suggests an upward trend between baseline and six weeks, and a downward trend between six and 26 weeks. Future research with a larger sample of schizophrenia spectrum and other psychotic disorders patients may shed more light on the possible association between IHT and self-efficacy in this population. More research is needed regarding the development and influence of self-efficacy for patients diagnosed with different disorders recovering from a mental health crisis.

## Strengths and limitations

The strengths of this study were the usage of data from an RCT, multiple time points of assessing self-efficacy and limited loss to follow-up. While previous studies relied primarily on routinely collected health data, we conducted interviews with patients experiencing a psychiatric crisis. There are several limitations that have to be mentioned. First, the effect of the intervention on self-efficacy was a pre-planned secondary outcome measure of the RCT [25]. That means that the sample size calculation was not powered to test the hypothesis regarding self-efficacy. More participants with personality disorders should have been included to have sufficient power to detect differences in self-efficacy. Second, the baseline measurement took place, on average, 3 weeks after the initiated crisis; this time period could be argued to be too long to measure the severity of the psychiatric crisis and therefore cause an underestimation of symptomatic recovery measured by the BPRS. Lastly, some of the psychiatric disorders, mainly personality disorders, had a small sample size. Conclusions regarding self-efficacy and the different mental health diagnoses should be considered with some caution.

## Conclusion and implications for practice

Contrary to our expectations, IHT did not provide an added advantage above CAU concerning improvement of self-efficacy in patients treated for a severe psychiatric crisis. In our sample of patients with a severe psychiatric crisis, we found that specific psychiatric disorders had a differential effect on the development of self-efficacy over time. Our study confirmed the idea that self-efficacy is positively associated with symptomatic recovery over time. Due to the complexity of crisis care, health care professionals may hold off strengthening self-efficacy of patients until after the crisis. However, given the role that self-efficacy has on recovery, we still see a prospect of an add-on intervention that has a positive effect on self-efficacy earlier on in the resolution of psychiatric crisis treatment, even given the complexity of crisis treatment. Nevertheless, additional research is needed to understand the complexity of the self-efficacy concept and its role and development in psychiatric disorders.

## Supplementary Information

**Additional file 1: Figure S1.** The intensive home treatment randomised controlled study, selection flowchart.

**Additional file 2: Table S1.** Sensitivity analysis of intensive home treatment on self-efficacy. Outcome of self-efficacy dimensions during 26 weeks using linear mixed modelling analyses.

**Additional file 3: Table S2.** Psychiatric disorders and the development of self-efficacy across three time points. Psychiatric disorders include depressive, bipolar, personality and schizophrenia spectrum and other psychotic disorders. The baseline, 6 and 26 weeks follow-up measurements were used in the analyses.

**Additional file 4: Table S3.** Association between self-efficacy, clinical recovery and quality of life during 26 weeks. Included data according per-protocol analyses. Recovery defined as a symptomatic outcome was measured using the Brief Psychiatric Rating Scale (BPRS). The 5-dimensional EuroQol instrument (EQ-5D-5L) was used to assess quality of life.

## Data Availability

The datasets generated and/or analysed during the current study are not publicly available due to containment of information that could compromise the privacy of research participants, but are available from the corresponding author on reasonable request.

## Abbreviations

EQ-5D-5L: 5-Dimensional EuroQol instrument
BPRS: Brief Psychiatric Rating Scale
CAU: Care as usual
DSM: Diagnostic and Statistical Manual of Mental Disorders
IHT: Intensive Home Treatment
MHCS: Mental Health Confidence Scale
RCT: Randomised controlled trial
QoL: Quality of life
