# Supplementary material for: The effects of intensive home treatment on self-efficacy in patients recovering from a psychiatric crisis
Source: Int J Ment Health Syst. 2021 Jan 6;15:1. doi: 10.1186/s13033-020-00426-y (PMC7789166; doi:10.1186/s13033-020-00426-y)

The effects of intensive home treatment on self-efficacy in patients recovering from a psychiatric crisis.

Ansam Barakat 1*; Matthijs Blankers; Jurgen E Cornelis; Nick M Lommerse; Aartjan FT Beekman; Jack JM Dekker.

1) Arkin Mental Health Care, Department of Research, Klaprozenweg 111 1033 NN Amsterdam The Netherlands * Correspondence @: ansam.barakat@arkin.nl

SUPPLEMENTARY MATERIAL

Fig. 1 Study selection Flowchart

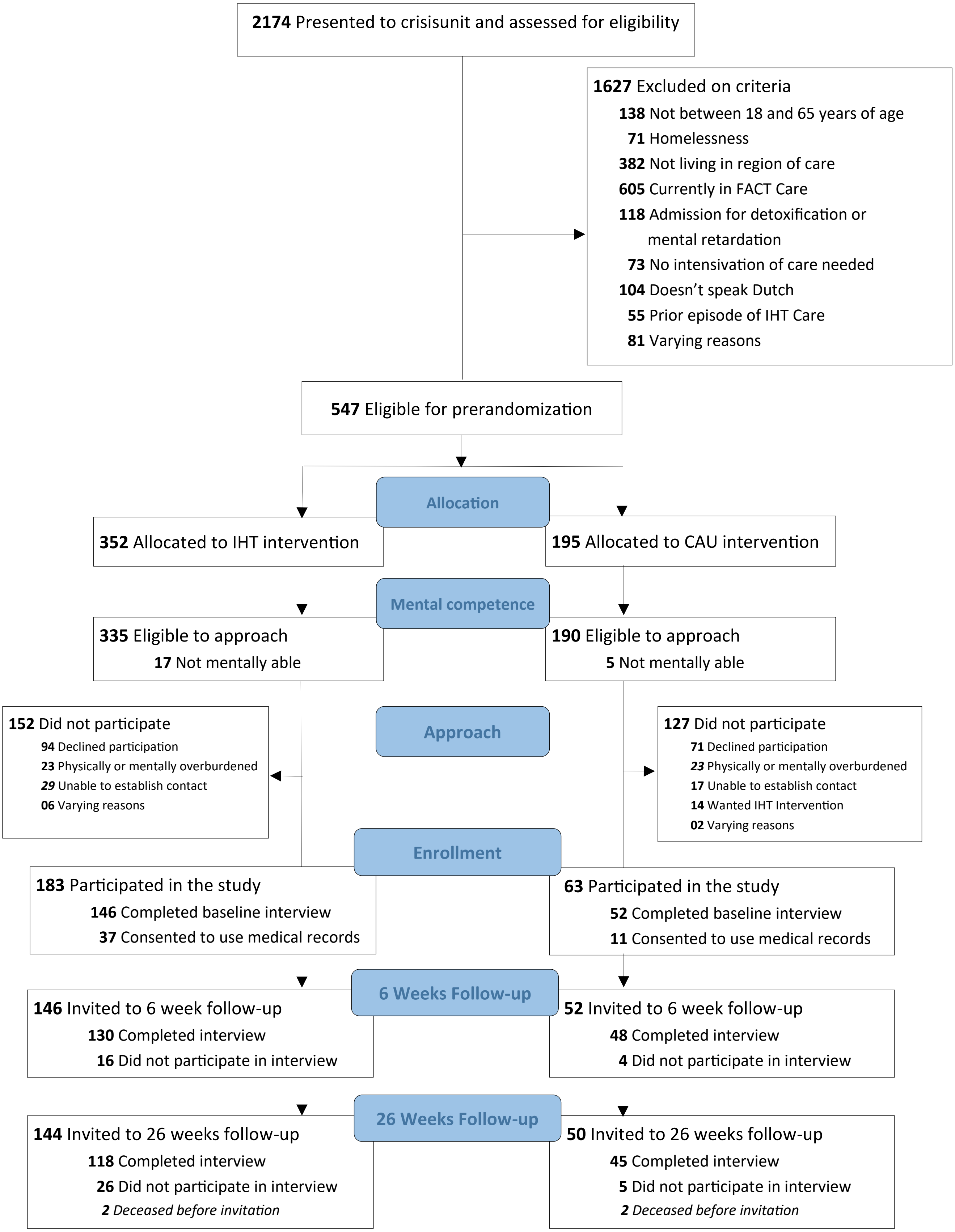

Supplement: Supplementary file 1 — Additional file 1: Figure S1. The intensive home treatment randomised controlled study, selection flowchart. [file 13033_2020_426_MOESM1_ESM.pdf]
