# Supplementary material for: The effects of intensive home treatment on self-efficacy in patients recovering from a psychiatric crisis
Source: Int J Ment Health Syst. 2021 Jan 6;15:1. doi: 10.1186/s13033-020-00426-y (PMC7789166; doi:10.1186/s13033-020-00426-y)
Supplement: Supplementary file 2 — Additional file 2: Table S1. Sensitivity analysis of intensive home treatment on self-efficacy. Outcome of self-efficacy dimensions during 26 weeks using linear mixed modelling analyses. [file 13033_2020_426_MOESM2_ESM.docx]

**The effects of intensive home treatment on self-efficacy in patients recovering from a psychiatric crisis.**

Ansam Barakat ^1^*; Matthijs Blankers; Jurgen E Cornelis; Nick M Lommerse; Aartjan TF Beekman; Jack JM Dekker.

^1^ Arkin Mental Health Care, Department of Research, Klaprozenweg 111 1033 NN Amsterdam The Netherlands

* Correspondence to Ansam Barakat, @: ansam.barakat@arkin.nl. ORCID: 0000-0002-5947-5110

| **Table 1. Sensitivity analysis of intensive home treatment on self-efficacy** | | | | | | | | | |
| --- | --- | --- | --- | --- | --- | --- | --- | --- | --- |
|  | | | | | | | | | |
| **Linear mixed modelling analyses** | | | | | | | | | |
|  |  |  |  |  |  |  |  | **95% CI** | |
| **Self-efficacy dimensions** | **N** | **Main effects** | **B** | **SE** | **DF** | **t** | ***p*** | **Lower** | **Upper** |
| Optimism | 168 | Treatment | -0.03 | 0.15 | 275.64 | -0.22 | 0.83 | -0.33 | 0.27 |
|  |  | Time | -0.04 | 0.15 | 155.26 | -0.29 | 0.77 | -0.34 | 0.25 |
|  |  | Time*Treatment | -0.08 | 0.18 | 155.95 | -0.44 | 0.66 | -0.42 | 0.27 |
|  |  | Intercept | 2.11 | 0.22 | 197.79 | 9.50 | <0.001 | 1.67 | 2.55 |
|  |  | Baseline Optimism | 0.52 | 0.04 | 163.27 | 12.10 | <0.001 | 0.44 | 0.61 |
| Advocacy | 168 | Treatment | -0.09 | 0.14 | 291.83 | -0.63 | 0.53 | -0.37 | 0.19 |
|  |  | Time | 0.10 | 0.15 | 151.93 | 0.69 | 0.49 | -0.20 | 0.41 |
|  |  | Time*Treatment | -0.01 | 0.18 | 152.78 | -0.05 | 0.96 | -0.36 | 0.34 |
|  |  | Intercept | 2.92 | 0.23 | 190.29 | 12.67 | <0.001 | 2.47 | 3.38 |
|  |  | Baseline Advocacy | 0.39 | 0.05 | 157.22 | 8.65 | <0.001 | 0.30 | 0.48 |
| Coping | 167 | Treatment | 0.00 | 0.16 | 258.25 | -0.03 | 0.98 | -0.31 | 0.30 |
|  |  | Time | 0.07 | 0.14 | 154.60 | 0.48 | 0.63 | -0.21 | 0.35 |
|  |  | Time*Treatment | -0.08 | 0.17 | 154.83 | -0.48 | 0.63 | -0.41 | 0.25 |
|  |  | Intercept | 2.12 | 0.21 | 198.94 | 9.96 | <0.001 | 1.70 | 2.54 |
|  |  | Baseline Coping | 0.50 | 0.05 | 168.55 | 11.05 | <0.001 | 0.41 | 0.59 |
| Self-efficacy | 167 | Treatment | -0.04 | 0.13 | 269.48 | -0.34 | 0.74 | -0.29 | 0.21 |
|  |  | Time | 0.02 | 0.12 | 153.98 | 0.13 | 0.89 | -0.22 | 0.26 |
|  |  | Time*Treatment | -0.05 | 0.14 | 154.68 | -0.36 | 0.72 | -0.33 | 0.23 |
|  |  | Intercept | 2.10 | 0.20 | 192.40 | 10.74 | <0.001 | 1.71 | 2.48 |
|  |  | Baseline Self-efficacy | 0.53 | 0.04 | 164.84 | 12.98 | <0.001 | 0.45 | 0.61 |
| *Note*. Self-efficacy = the total of all self-efficacy dimensions. B = estimated regression coefficient. SE= Standardised Error. DF = degrees of freedom. t = T-test value. CI = Confidence Interval. The outcome based on intension to treat analysis. | | | | | | | | | |

**Additional file**
