# Supplementary material for: The effects of intensive home treatment on self-efficacy in patients recovering from a psychiatric crisis
Source: Int J Ment Health Syst. 2021 Jan 6;15:1. doi: 10.1186/s13033-020-00426-y (PMC7789166; doi:10.1186/s13033-020-00426-y)
Supplement: Supplementary file 3 — Additional file 3: Table S2. Psychiatric disorders and the development of self-efficacy across three time points. Psychiatric disorders include depressive, bipolar, personality and schizophrenia spectrum and other psychotic disorders. The baseline, 6 and 26 weeks follow-up measurements were used in the analyses. [file 13033_2020_426_MOESM3_ESM.docx]

**The effects of intensive home treatment on self-efficacy in patients recovering from a psychiatric crisis.**

Ansam Barakat 1*; Matthijs Blankers; Jurgen E Cornelis; Nick M Lommerse; Aartjan TF Beekman; Jack JM Dekker.

^1^ Arkin Mental Health Care, Department of Research, Klaprozenweg 111 1033 NN Amsterdam The Netherlands

* Correspondence to Ansam Barakat, @: ansam.barakat@arkin.nl. ORCID: 0000-0002-5947-5110

**Additional file**

| **Table 2. Psychiatric disorders and the development of self-efficacy across three time points** | | | | | | | | |
| --- | --- | --- | --- | --- | --- | --- | --- | --- |
| **Mental health disorders** | **(I) Time** | **(J) Time** | **Mean Difference (I-J)** | **SE** | **DF** | ***p*** | **95% CI for Difference ^c^** | |
|  |  |  |  |  |  |  | **Lower** | **Upper** |
| Depressive disorders | Baseline | 6 weeks | -0.69 | 0.15 | 221.89 | <0.001 | -1.06 | -0.32 |
|  |  | 26 weeks | -0.64 | 0.16 | 226.18 | <0.001 | -1.02 | -0.26 |
|  | 6-weeks | Baseline | 0.69 | 0.15 | 221.89 | <0.001 | 0.32 | 1.06 |
|  |  | 26 weeks | 0.05 | 0.15 | 217.48 | 1.00 | -0.32 | 0.42 |
| Bipolar disorders | Baseline | 6 weeks | 0.19 | 0.15 | 226.23 | 0.65 | -0.18 | 0.56 |
|  |  | 26 weeks | 0.29 | 0.15 | 223.92 | 0.17 | -0.07 | 0.66 |
|  | 6-weeks | Baseline | -0.19 | 0.15 | 226.23 | 0.65 | -0.56 | 0.18 |
|  |  | 26 weeks | 0.11 | 0.15 | 215.44 | 1.00 | -0.26 | 0.47 |
| Schizophrenia Spectrum and Other Psychotic disorders | Baseline | 6 weeks | -0.12 | 0.14 | 221.46 | 1.00 | -0.46 | 0.22 |
|  |  | 26 weeks | 0.06 | 0.15 | 219.56 | 1.00 | -0.31 | 0.42 |
|  | 6-weeks | Baseline | 0.12 | 0.14 | 221.46 | 1.00 | -0.22 | 0.46 |
|  |  | 26 weeks | 0.18 | 0.15 | 223.97 | 0.73 | -0.19 | 0.55 |
| Personality disorders | Baseline | 6 weeks | -0.32 | 0.28 | 214.45 | 0.77 | -1.00 | 0.36 |
|  |  | 26 weeks | -0.72 | 0.29 | 217.79 | 0.04 | -1.43 | -0.02 |
|  | 6-weeks | Baseline | 0.32 | 0.28 | 214.45 | 0.77 | -0.36 | 1.00 |
|  |  | 26 weeks | -0.40 | 0.28 | 214.45 | 0.46 | -1.08 | 0.28 |
| *Note.* Included data according per-protocol analyses, based on estimated marginal means. SE = Standardised Error. DF = degrees of freedom. CI = Confidence Interval. c = adjustment for multiple comparisons using the Bonferroni corrections. | | | | | | | | |
