# Supplementary material for: The effects of intensive home treatment on self-efficacy in patients recovering from a psychiatric crisis
Source: Int J Ment Health Syst. 2021 Jan 6;15:1. doi: 10.1186/s13033-020-00426-y (PMC7789166; doi:10.1186/s13033-020-00426-y)
Supplement: Supplementary file 4 — Additional file 4: Table S3. Association between self-efficacy, clinical recovery and quality of life during 26 weeks. Included data according per-protocol analyses. Recovery defined as a symptomatic outcome was measured using the Brief Psychiatric Rating Scale (BPRS). The 5-dimensional EuroQol instrument (EQ-5D-5L) was used to assess quality of life. [file 13033_2020_426_MOESM4_ESM.docx]

| **The effects of intensive home treatment on self-efficacy in patients recovering from a psychiatric crisis.**  Ansam Barakat ^1^*; Matthijs Blankers; Jurgen E Cornelis; Nick M Lommerse; Aartjan TF Beekman; Jack JM Dekker.  ^1^ Arkin Mental Health Care, Department of Research, Klaprozenweg 111 1033 NN Amsterdam The Netherlands  * Correspondence to Ansam Barakat, @: ansam.barakat@arkin.nl. ORCID: 0000-0002-5947-5110  **Additional file** | | | | | | | | | |
| --- | --- | --- | --- | --- | --- | --- | --- | --- | --- |
| **Table 3. Association between self-efficacy, clinical recovery and quality of life during 26 weeks** | | | | | | | | | |
|  |  |  |  |  |  |  |  | **95% CI** | |
|  | **N** | **Main effects** | **B** | **SE** | **DF** | **t** | ***p*** | **Lower** | **Upper** |
| **Symptomatic recovery** | | | | | | | | | |
| Total BPRS | 139 | Intercept | 2.15 | 0.09 | 351.91 | 22.98 | <0.001 | 1.97 | 2.34 |
|  |  | Self-efficacy | -0.10 | 0.02 | 351.36 | -4.66 | <0.001 | -0.15 | -0.06 |
|  |  | Time | 0.11 | 0.07 | 257.84 | 1.57 | 0.12 | -0.03 | 0.24 |
|  |  | Time*Self-efficacy | -0.06 | 0.02 | 257.49 | -3.71 | <0.001 | -0.09 | -0.03 |
| Positive symptoms | 139 | Intercept | 1.08 | 0.19 | 353.91 | 5.81 | <0.001 | 0.71 | 1.44 |
|  |  | Self-efficacy | 0.14 | 0.04 | 354.91 | 3.22 | <0.001 | 0.06 | 0.23 |
|  |  | Time | 0.38 | 0.14 | 271.33 | 2.65 | 0.01 | 0.10 | 0.66 |
|  |  | Time*Self-efficacy | -0.15 | 0.03 | 271.52 | -4.50 | <0.001 | -0.22 | -0.09 |
| Negative symptoms | 139 | Intercept | 1.69 | 0.10 | 195.58 | 17.56 | <0.001 | 1.50 | 1.88 |
|  |  | Self-efficacy | -0.11 | 0.02 | 195.30 | -4.75 | <0.001 | -0.15 | -0.06 |
|  |  | Time | -0.10 | 0.06 | 144.37 | -1.72 | 0.09 | -0.22 | 0.02 |
|  |  | Time*Self-efficacy | 0.02 | 0.01 | 146.10 | 1.37 | 0.17 | -0.01 | 0.05 |
| Depression and Anxiety | 139 | Intercept | 4.77 | 0.20 | 353.95 | 23.73 | <0.001 | 4.38 | 5.17 |
|  |  | Self-efficacy | -0.55 | 0.05 | 351.94 | -11.72 | <0.001 | -0.65 | -0.46 |
|  |  | Time | 0.04 | 0.14 | 258.05 | 0.27 | 0.79 | -0.25 | 0.32 |
|  |  | Time*Self-efficacy | -0.04 | 0.03 | 257.56 | -1.19 | 0.24 | -0.11 | 0.03 |
| Disorganisation | 139 | Intercept | 1.28 | 0.11 | 350.77 | 11.40 | <0.001 | 1.06 | 1.50 |
|  |  | Self-efficacy | 0.06 | 0.03 | 351.94 | 2.15 | 0.03 | 0.00 | 0.11 |
|  |  | Time | 0.14 | 0.08 | 259.56 | 1.60 | 0.11 | -0.03 | 0.30 |
|  |  | Time*Self-efficacy | -0.07 | 0.02 | 259.60 | -3.38 | <0.001 | -0.11 | -0.03 |
| **Quality of Life** | | | | | | | | | |
| EQ-5D-5L | 139 | Intercept | 0.20 | 0.06 | 353.99 | 3.25 | <0.001 | 0.08 | 0.32 |
|  |  | Self-efficacy | 0.14 | 0.01 | 352.50 | 9.59 | <0.001 | 0.11 | 0.17 |
|  |  | Time | 0.02 | 0.05 | 265.48 | 0.39 | 0.69 | -0.07 | 0.11 |
|  |  | Time*Self-efficacy | <0.001 | 0.01 | 264.27 | -0.34 | 0.73 | -0.03 | 0.02 |
| *Note.* Included data according per-protocol analyses, based on estimated marginal means. BPRS = the Brief Psychiatric Rating Scale. B = estimated regression coefficient. SE = Standardised Error. DF = degrees of freedom. t = T-test value. CI = Confidence Interval. EQ-5D-5L = 5-dimensional EuroQol instrument. | | | | | | | | | |
